# A New Hope: Can we estimate a clinical diagnosis by using eye-tracking technology?

**DOI:** 10.1192/j.eurpsy.2025.1675

**Published:** 2025-08-26

**Authors:** M. Alijotas Capdevila, M. Martínez-Ramirez, P. Querol Clares, I. Setién Ramos, J. Lugo Marin, J. A. Ramos-Quiroga, L. Gisbert Gustemps

**Affiliations:** 1 Psychiatry; 2 Neuropsychology; 3 Psychology, Hospital Vall d’Hebron, Barcelona, Spain

## Abstract

**Introduction:**

Eye-tracking technology is a sensitive and direct method of measuring social-cognitive abilities regardless of language, causing minimal cognitive strain and with the advantage of its passive presentation of stimuli to participants, which allows observation without requiring verbal interaction nor explicit responses.

**Objectives:**

Our aim was to study variations in social attention among adults diagnosed with ASD, ADHD, comorbid ASD and ADHD, and non-clinical individuals using eye-tracking technology.

**Methods:**

We recruited a total of 80 participants over the age of 18 from the Psychiatric External Ward of Vall Hebron University Hospital, resulting in approximately 20 participants in each group. Social attention was assessed using vignettes from the Autism Diagnostic Observation Schedule-2 (ADOS-2).

**Results:**

Preliminary results revealed significant statistical differences among the groups in the variables analyzed, indicating that individuals with ASD display distinct patterns of visual attention toward Areas of Interest (AOIs) containing social information compared to the other groups. No differences were found in the duration of fixation on the characters’ eyes among the groups. However, participants diagnosed with ASD and ASD+ADHD tended to spend more time looking at regions with lower social information density, such as the characters’ hands in the third vignette.

**Conclusions:**

These findings shed light on social attention profiles across clinical and non-clinical populations, indicating specific patterns associated particularly with ASD, and highlight the importance of considering multiple aspects of social cognition. By employing eye-tracking technology, this study provides valuable insights into the underlying mechanisms of social attention across different neurodevelopmental conditions. Furthermore, the lack of significant differences in eye fixation duration on the eyes among clinical groups challenges conventional beliefs regarding social attention deficits in ADHD compared to ASD.

Using objective measures like eye-tracking can enhance our understanding of these complex neurodevelopmental conditions, potentially aiding in diagnosis and guiding the development of targeted interventions to address social cognition challenges in populations with neurodevelopmental disorders.

Our findings may contribute to a better understanding of social attention differences between ASD, ADHD, comorbid ASD+ADHD, and non-clinical individuals, paving the way for the design of more tailored and effective interventions.

**Disclosure of Interest:**

None Declared

